# ESR Study of Interfacial Hydration Layers of Polypeptides in Water-Filled Nanochannels and in Vitrified Bulk Solvents

**DOI:** 10.1371/journal.pone.0068264

**Published:** 2013-06-28

**Authors:** Yei-Chen Lai, Yi-Fan Chen, Yun-Wei Chiang

**Affiliations:** 1 Department of Chemistry and Frontier Research Center on Fundamental and Applied Sciences of Matters, National Tsing Hua University, Hsinchu, Taiwan; 2 Department of Chemical and Materials Engineering, National Central University, Jhongli, Taiwan; Jacobs University Bremen, Germany

## Abstract

There is considerable evidence for the essential role of surface water in protein function and structure. However, it is unclear to what extent the hydration water and protein are coupled and interact with each other. Here, we show by ESR experiments (cw, DEER, ESEEM, and ESE techniques) with spin-labeling and nanoconfinement techniques that the vitrified hydration layers can be evidently recognized in the ESR spectra, providing nanoscale understanding for the biological interfacial water. Two peptides of different secondary structures and lengths are studied in vitrified bulk solvents and in water-filled nanochannels of different pore diameter (6.1∼7.6 nm). The existence of surface hydration and bulk shells are demonstrated. Water in the immediate vicinity of the nitroxide label (within the van der Waals contacts, ∼0.35 nm) at the water-peptide interface is verified to be non-crystalline at 50 K, and the water accessibility changes little with the nanochannel dimension. Nevertheless, this water accessibility for the nanochannel cases is only half the value for the bulk solvent, even though the peptide structures remain largely the same as those immersed in the bulk solvents. On the other hand, the hydration density in the range of ∼2 nm from the nitroxide spin increases substantially with decreasing pore size, as the density for the largest pore size (7.6 nm) is comparable to that for the bulk solvent. The results demonstrate that while the peptides are confined but structurally unaltered in the nanochannels, their surrounding water exhibits density heterogeneity along the peptide surface normal. The causes and implications, especially those involving the interactions between the first hydration water and peptides, of these observations are discussed. Spin-label ESR techniques are proven useful for studying the structure and influences of interfacial hydration.

## Introduction

Water is essential for the stability and function of biological macromolecules, such as proteins, and DNA. Recent studies have investigated water in the first few hydration layers of proteins, the so-called “biological surface water”. [1]–[3] Evidence has been gathered to suggest that the properties of this surface water shell are extremely important to biological functioning of a protein; [4], [5] on the contrary, some studies have reported weak interdependence between the interfacial hydration and the protein structure, where the later is of tremendous influence to its functioning. [6], [7] The role of the interfacial hydration therefore remains a matter of debate. To resolve this ambiguity, a molecular scale picture of how water and protein structurally and functionally interact with each other, and why the first surface hydration water differs from bulk water is much needed but still largely lacking. This absence severely impedes our understanding toward the biological water. A reliable tool that has sufficient sensitivity and accuracy to probe the changes, at a molecular level, in dynamics and structure for both protein and water molecules has been long desired. [1], [8] Recently, spin-label electron spin resonance (ESR) has been demonstrated useful for probing water dynamics. [9], [10] Built upon this progress, an approach combining ESR with nanochannels has emerged as a potentially useful tool for studying protein-water interactions; it demonstrated that the nanochannel of mesoporous materials was useful to maintain an amorphous state of solvents, which is required for studying protein structures and their dependence on water properties. [11], [12] Moreover, studying the biological surface water within nanochannels *per se* is also of fundamental importance in nature, since it is related to the origins of life. [13] Here, we report a carefully comparative investigation on the surface water molecules that directly interact with the biomolecules in nanochannels versus those for bulk solvents, and demonstrate that the surface hydration layer can be evidently recognized in the ESR spectroscopy.

In this work, we studied the structural conformations and local solvation of a 26-residue polypeptide (n3) and a 14-proline-long model peptide (PPm3), as immersed in bulk solvents versus encapsulated within the nanochannels of mesoporous materials, by several of the pulsed ESR techniques including double electron-electron resonance (DEER), electron spin echo (ESE), and electron spin echo envelope modulation (ESEEM) experiments. The n3 and PPm3 peptides in pure water have a propensity to a β-hairpin structure and a left-handed polyproline II (PPII) helix conformation, respectively. In the spin-label ESR studies, [14]–[16] the introduction of a cysteine-specific paramagnetic nitroxide probe is accomplished through cysteine substitution mutagenesis; for peptide, cysteine is introduced during synthesis. The DEER technique [17], [18] allows a careful examination of the differences in the biomolecular structures before/after the encapsulation into the nanochannels. [11] The ESE and ESEEM are two powerful tools capable of reporting the coupling between a nitroxide spin and nearby water protons within the ranges of ∼2 nm and ∼0.6 nm, respectively. [19], [20]. We show by DEER technique that the two peptides retain their secondary structures after the encapsulation into the nanochannels as well as in a mostly vitrified bulk solvent at cryogenic temperatures. In the further ESE and ESEEM measurements, we carefully examine the density of the interfacial water within the range of 2 nm from the peptide surface. The inner (i.e., the first hydration shell) and outer (the bulk-like shell) water shells respond differently regarding the nanochannel diameters and with/without nanoconfinement. The differences are quantitatively described by the water density derived from the ESE and ESEEM measurements. The details regarding the local structural changes of the water/peptide molecules are thus unambiguously revealed based on the results of the ESR spectroscopy.

## Materials and Methods

### Polypeptides

**Figure 1:**
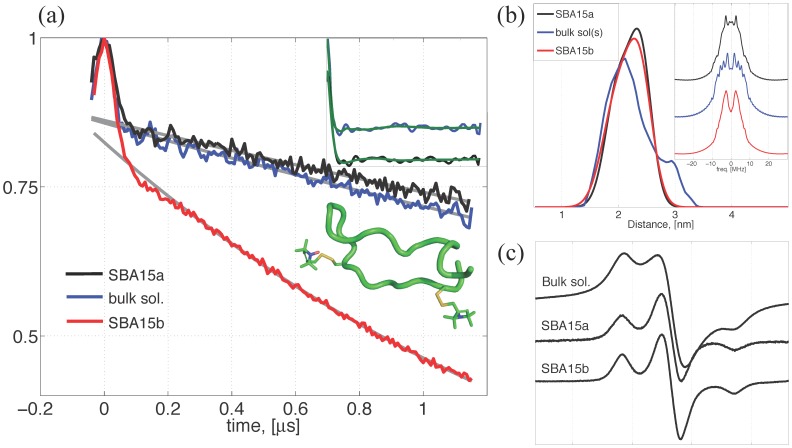
Determination of the n3β-d structure. (a) The time-domain DEER data for the n3β-d (0.5 mM) in the studied conditions, including the vitrified bulk solvent (sol(s)/H_2_O) and the nanochannels (SBA15a and SBA15b). The gray lines represent the exponential baselines that best fit the DEER data. There are two insets. One displays a ribbon model for the n3β-d showing the spin-label side-chains at the 3rd and 9th sites of the peptide. The model was derived from a NMR study (PDB code: 1G04). The other inset shows the baseline-corrected DEER traces for the sol(s)/H_2_O and the SBA15a, and also the simulated DEER traces (in green color) using the obtained P(r)s. There are some distinct differences in the two traces. (b) The (normalized) interspin distance distributions of the n3β-d peptides in the conditions studied. The average distances of the three measurements are approximately the same, indicating the n3β structure remains roughly unchanged. A much-broadened P(r) for the bulk solution study is obtained due to the solvent heterogeneity. The inset shows the Pake doublets converted from the DEER data. (c) Cw-ESR spectra of the n3β-d at 50 K. The clustering, caused by the solvent heterogeneity at 50 K, is evidently observed in the cw-ESR spectra of the bulk solution study, but not in the nanochannel studies.

**Figure 2:**
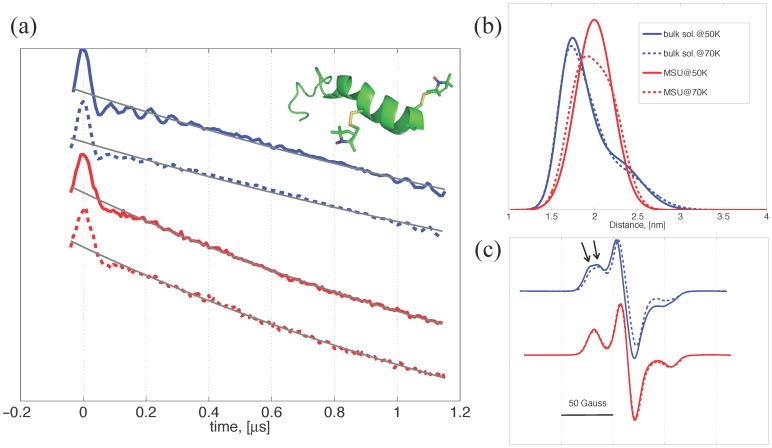
Determination of the n3α-d structure. (a) The time-domain DEER signals of the studied conditions. The gray lines represent the exponential baselines that best fit the data. Inset shows a ribbon model of the n3α-d derived from NMR data (PDB code: 1M25). (b) The P(r) distributions extracted from the time-domain DEER data by the Tikhonov regularization analysis. The average distances (∼2.0 nm) are consistent with the expectation. (c) The cw-ESR spectra of the n3α-d. The spectra of the bulk solution studies are characterized by a broader linewidth and the spectral heterogeneity (indicated by arrows) as compared to the spectra of the nanochannel studies.

All peptides of this study were custom-synthesized by Kelowna International Scientific Inc. (New Taipei, Taiwan) with purity greater than 95%. The n3 peptide is a linear 26-mer polypeptide with a sequence of GNDYEDRYYRENMYRYPNQVYYRPVA, where the first 25 residues correspond to the domain 142–166 of the human prion protein and the underlined letters represent the segments that correspond to helix H1 and β-strand S2, respectively, in the normal prion structure. [21] It is highly soluble and exhibits an intrinsic propensity to a β-hairpin conformation at neutral pH in the PB buffer (10 mM sodium phosphate, pH 6.5) or in pure water. [12] It was previously demonstrated that the n3 peptide folds into an α-helical conformation in a TFE(trifluoroethanol)/PB buffer. [12], [22] Two mutations of the n3 peptide were studied. They were substituted with a cysteine at the 9th site and at both the 3rd and 9th sites, respectively, and were denoted as n3-s and n3-d (cf. Figures 1a and 2a for the structures of the n3β-d and n3α-d, respectively). Pure H_2_O and D_2_O were used in the n3-s study by ESEEM. The PPm3-s is a 14-mer-long polyproline model peptide, with the 9th residue substituted with cysteine. Previously this PPm3-s was shown to remain a PPII helical structure as immersed in a vitrified bulk solvent (40% sucrose in water) or encapsulated into the mesoporous materials containing pure water.

The circular dichroism (CD) spectroscopy measurements for the n3-s and n3-d peptides as in the nanochannels versus in the bulk solvent (at 4°C and 25°C) were previously reported in Huang et al [12] and shown to resemble each other closely showing a typical β-hairpin structure (or an α-helical structure in the TFE/PB solvent). The results indicated that the secondary structures of the studied n3 peptide variants remain unchanged over the investigated experimental conditions [23].

### Nanochannels

The mesoporous silica SBA15 materials were synthesized as previously described. [24] Briefly, tetraethoxysilane was added to the HCl solution of triblock copolymer Pluronic P-123 (EO_20_PO_70_EO_20_). The molar composition was 1 TEOS : 0.54 HCl : 100 H_2_O : 0.017 P-123. The mixture was stirred at 35°C for 24 h, aged at 90°C (SBA15a) and 60°C (SBA15b), for 24 h and then filtered and dried. The copolymer molecules in the as-synthesized SBA15 samples were removed by treating the samples with concentrated sulfuric acid at 90°C followed by calcination at 350°C. The structural properties of the mesoporous materials are summarized in the following order: SBA15a, SBA15b. The pore diameters are 7.6 and 6.1 nm. The unit cell sizes are 11.6 and 9.6 nm. The sizes of the wall thickness are thus 4.0 and 3.5 nm. The nanochannel used for the n3α-d study was a mesoporous silica material with a cross-linked hexagonal pore structure and an average pore size of 7.1 nm (known as MSU), and was purchased from Aldrich. The MSU nanochannel was demonstrated useful for encapsulating spin-labeled peptides for ESR study. [11] The smallest pore size (6.1 nm) for the DEER measurements of the present study is at least 1.5 times larger than the globular sizes of the n3 and PPm3 peptides. In DEER measurements, different nanochannels would result in a change in the slope of the signal baseline, but give no rise to changes in the secondary structures.

### Experimental Procedures

In the spin-labeling experiment, peptides were labeled with a 10-fold excess of (1-Oxy-2,2,5,5-tetramethyl-3-pyrroline-3-methyl) methanethiosulfonate spin label (MTSL) (Alexis biochemicals, San Diego, CA) per cysteine residue for overnight in the dark at 4°C. They were further purified by reverse phase HPLC as previously described. [12] MALDI-TOF experiments were conducted to confirm the identity of the peptides carrying the spin labels. In the bulk solution studies, two cryoprotectants (sucrose and glycerol) were used. The concentration of the spin-labeled peptides was 0.5 mM for n3β-d; The concentration was 1.5 mM for the studies of n3β-s and PPm3-s. We used the n3-d in the DEER measurements and n3-s as well as PPm3-s in the ESEEM and ESE measurements. For the n3α studies, the concentration of the n3α-d was 0.9 mM in the (mostly) vitrified solution of the 40% (w/w) sucrose and 87/13 (v/v) TFE/PB mixture. The n3α-d concentration for the nanochannel study was 1.2 mM in the solvent of 87/13 (v/v) TFE/PB. The solution volume added into an ESR tube for the bulk solution studies was ca. 40 µL. A higher percentage of the cryoprotectant in solution was rarely reported, and was not recommended for biological experiments as it could easily cause disturbance to the native conformation of biomolecules. The encapsulation of the samples into the nanochannels was prepared as previously described. [11], [12], [25] The preparation approach has proven useful for effectively trapping the added solution within the nanochannels. The solution volume added onto the mesoporous materials (0.1 mL; 12 mg) was ca. 20 µL. After the encapsulation, the outside surface of the nanochannel materials appeared dried. No cryoprotectant was used for the nanochannel experiments. To perform the deuterium-hydrogen exchange experiment to modify the surface group of the nanochannel materials, a previously developed procedure was followed. [26] The materials were placed in a vacuum oven at 100°C overnight to remove moisture. Dried mesoporous materials were submerged into pure excess D_2_O at room temperature. After >1.5 hrs, the excess D_2_O was pipetted out and the sample was refreshed with fresh D_2_O. The cycle was repeated four times, followed by an overnight incubation. The sample was dried again before the peptide encapsulation.

In summary of the studied solvent conditions, sol(s)/H_2_O represents a mixture of a bulk solvent containing 40 wt% sucrose and H_2_O; as D_2_O is used, it is denoted by sol(s)/D_2_O. Sol(g)/H_2_O stands for a mixture of a bulk solvent containing 40v/v% glycerol and H_2_O, while sol(dg)/D_2_O denotes a mixture of a bulk solvent containing 40v/v% deuterated glycerol and D_2_O.

### Cw/Pulsed ESR Measurements and Data Analysis

A Bruker ELEXSYS E580 cw/pulsed spectrometer, equipped with a Bruker pulse ELDOR unit E580-400, a dielectric resonator (ER4118X-MD5W), and a helium gas flow system (4118CF and 4112HV), was used. DEER experiments were carried out using the four-pulse constant-time DEER sequence: π/2(ω_A_)−τ_1_−π(ω_A_)−(τ_1_+t)− π(ω_B_)−(τ_2_−t)−π(ω_A_)−τ_2_−echo. The detection pulses (ω_A_) were set to 32 and 16 ns for π and π/2 pulses, respectively and pump frequency (ω_B_) was set to approximately 70 MHz lower than the detection pulse frequency. The pulse amplitudes were chosen to optimize the refocused echo. The π/2-pulse was employed with +x/−x phase cycle to eliminate receiver offsets. The duration of the pumping pulse was about 32 ns, and its frequency (ω_B_) was coupled into the microwave bridge by a commercially available setup from Bruker. All pulses were amplified via a pulsed traveling wave tube amplifier (E580–1030). The field was adjusted such that the pump pulse is applied to the maximum of the nitroxide spectrum, where it selects the central m_I_  = 0 transition of A_zz_ together with the m_I_ = ±1 transitions. The echo was measured as a function of t, while τ_2_ was kept constant, depending on T_M_. Typical numbers of shots per points and scan number were set to 1024 and 30–40, respectively. Accumulation time for each set of data was about 12 hours. Most of the pulsed ESR experiments of this study were performed at 50 K while some were performed at 70 K. The employed cooling scheme is described below. The determination of interspin distance distribution of the DEER data was performed in the time-domain analysis by Tikhonov regularization based on the L-curve method. [27], [28] This four-pulse DEER experiment has become a widely used approach for measuring distances between electron spins (or spin labels) in proteins and protein/membrane systems in the range approximately 1.5 to 8 nm. [29] The cw-ESR experiment was performed at an operating frequency of 9.4 GHz and 1.5 mW incident microwave power. The swept magnetic range was 200 Gauss.

The three-pulse ESEEM is a powerful technique to measure weak coupling between paramagnetic ions and nearby (<0.6 nm) nuclei with nonzero spin metals in biological systems, such as metalloproteins and bioinorganic compounds. [20], [30] Its sequence was π/2–τ–π/2–T–π/2–τ–Stimulated Echo with a pulse length t_π/2_ = 16 ns, the T value starting from 400 ns with a time increment ΔT  = 4 ns. The data were collected by monitoring the amplitude of the stimulated echo as the time T was increased by ΔT. In order to avoid the blind spot and to ensure that all frequencies are being detected, signals were collected by varying the time τ from 100 to 400 ns. The τ values of 260 and 100 ns were selected in the τ variation experiments and used for three-pulse ESEEM measurements. A four-step phase cycling was used to remove unwanted echoes. The time-domain ESEEM data were first baseline corrected by a stretched exponential, zero filled and afterwards fast Fourier transformed. The FT-ESEEM spectra were shown in absolute magnitude. All the theoretical simulations and analyses were performed using MATLAB with EasySpin toolbox [31].

ESE is a useful tool to provide information concerning the interaction between an unpaired spin and neighboring electron and nuclear spins approximately within a 2-nm range. The ESE experiments were applied using the 2-pulse Hahn echo sequence, consisting of a π/2 pulse along the x-axis followed by a delay τ and a train of π pulses, separated by interpulse delays 2τ. [32] The field was adjusted to optimize the spin echo, and the duration times of π/2 and π pulses were set to 16 and 32 ns. As previously described, [11] the ESE signals were fitted to a stretched exponential function to extract T_M_ value from the ESE data.

A common cooling approach was used. The sample tube was plunge-cooled in liquid nitrogen, and then transferred into the ESR probehead, which was pre-cooled to 50 K using the helium flow system. This rapid cooling scheme is supposed to preserve the room-temperature molecular features of all relevant molecules at cryogenic temperatures, as premised in the X-ray protein cryo-crystallography [33]; the molecular conformations observed at cryogenic temperatures are thus *snapshots* of what had happened at room-temperature. [34], [35] Further discussion regarding this aspect is presented below. To test the consistency of the cooling procedure, some measurements (e.g. the n3 studies) were repeated three times, by cooling different samples taken from the same stock solution. Same cooling procedure was followed in all of the measurements to ensure that the cooling rate is approximately the same. The results were highly reproducible.

### Experimental Verifications of the Peptide Encapsulation and Preservation of the Peptide Properties upon Encapsulation

To verify the peptides are adequately encapsulated into the nanochannels, this study performed the following experiments. An excess buffer was added into the ESR tube containing the mesoporous materials, wherein the spin-labeled peptides were encapsulated as described above. The tube was sent for ESR measurements at room temperature. The collected spectra were found to remain identical to the spectra collected before the addition of the excess buffer; that is, the spectra show typical slow-motional lineshapes. This experiment demonstrated that the spin-labeled peptides were not adsorbed/left on the outer surface of the materials and that the molecules were trapped adequately well within the nanochannels. This study also confirmed that no ESR signal was obtained for the supernatant liquid after centrifugation.

In all of the nanochannel studies, no cryoprotectant was used. At such a low temperature (50 K), pure water became ice. In one control experiment, this study obtained noise-like signals in the pulsed ESE measurements for the n3β in the bulk water without the cryoprotectant. This control experiment supported the following. In the nanochannel experiments, the tiny amount of the solution that might be left outside the mesopores would become ice crystals, resulting in an extremely short spin phase memory time (T_M_). Accordingly, the rapidly decaying signals (due to the short T_M_ value) would by no means be observed in our pulsed ESR experiments. Therefore, the meaningful pulsed ESR signals that we collected at 50 K were absolutely from the samples within the nanochannels.

Previously, [11], [12], [25] we studied the n3α and n3β peptides by cw-ESR. We demonstrated that the spectra of the n3α and n3β peptides were distinctly different as confined in the nanochannels of the same material, and that the spectra varied reasonably concerning temperature variations (200 ∼ 300 K). Besides, it was confirmed by the CD and Pulsed ESR experiments that the secondary structures of the n3 peptides were not disrupted in the nanochannels. [12] Same observations were made for the PPm3, showing that the PPm3 retained its PPII structure in the nanochannels. [23] These observations evidently indicated that the interaction between spin label and the inner surface of the nanochannel, if any, would definitely be insignificant as compared to the backbone dynamics of the secondary structures and the interactions between solvent molecules and the peptides. Otherwise, the spectra of the n3 peptides in helical versus hairpin forms would have appeared similar. In view of the observations of the experiments described above, it is shown that the peptides are immersed in solvent and are confined but free as encapsulated within the nanochannels.

## Results

### Determination of n3β Peptide Structure by DEER

Two different nanochannels, SBA15a and SBA15b with pore diameters of 7.6 and 6.1 nm, respectively, are used. In the bulk solution experiments, the (mostly) vitrified bulk solvent is 40 wt% sucrose in H_2_O (or D_2_O), denoted by sol(s)/H_2_O or sol(s)/D_2_O. In the nanochannel experiments, the solvent contains no cryoprotectant (sucrose).

The time-domain DEER traces for the three studied conditions (with the same nominal spin concentration of 0.5 mM) are shown in Figure 1(a), wherein a cartoon model of the n3β-d peptide is shown as an inset. The baseline for the SBA15b decays rapidly as compared to the baselines of the SBA15a and bulk solution studies, indicating that the bulk spin concentration is increased upon the encapsulation into the SBA15b. The effect of the increased concentration in the SBA15b will be discussed later. A typical DEER analysis procedure was performed to remove the baselines with a homogeneous model. [36] As the time-domain traces for the sol(s)/H_2_O (blue) and SBA15a (black) look somewhat similar in Figure 1(a), we plot their respective normalized baseline-corrected DEER traces in the inset (upper right) of Figure 1(a), wherein the green lines represent the simulated DEER traces using the P(r) shown in Figure 1(b). The two traces in the inset are shifted by 0.05 in y-axis for clarity. The inset plot clearly shows that there are small but significant differences in the two traces. Figure 1(b) reports the intra-molecular interspin distance distributions P(r), determined by the Tikhonov analysis, [27] for the DEER time-domain data after the baseline removal. The P(r) results are normalized to have the same area. The two distance distributions of the nanochannel studies are alike, showing the same average distance of ∼2.2 nm. It is consistent with the structure and backbone flexibility of the n3 peptide derived from the NMR data taken at 5–37 °C. [21] Conducted at 200∼300K, a previous study demonstrated that the side-chain disordering (i.e., the cone width of the side-chain) of the spin-labeled n3, as confined in nanochannels, decreased substantially as compared to the disordering in the bulk solvent. [12] Therefore, the obtained P(r) results of the confined n3β-d largely reflect the backbone conformation and flexibility that are closely correlated with the local solvent heterogeneity (although the contribution from the side-chain rotamers cannot be completely excluded). For the bulk solution study, the result shows a most probable distance around 2.1 nm and a minor peak at ∼3 nm. This heterogeneous distribution is in accordance with a previous finding [11] showing a broadened trimodal-like P(r), with peaks at 2.0, 2.2, and 2.9 nm, for the same solvent and the same n3β-d peptide at 50 K. Taken together, this heterogeneity in the P(r) for the n3β-d can be consistently observed for the bulk solvent case in different measurements. To confirm the P(r) results, we perform Fourier transformation to convert the DEER data to Pake doublets as shown in inset of Figure 1(b). The splitting of the Pake doublets is distinctly less obvious in the bulk solution data than in the nanochannel results, validating the existence of the 3-nm distance peak in the P(r) of the bulk solution study. Moreover, this study verified the existence of the 3-nm peak with a newly developed method [37] and confirmed that the peak is not a ghost peak and cannot be removed by adding noise to the time-domain data. The cw-ESR spectra collected at 50 K (Figure 1c) shows that a large spectral broadening, which is caused by the peptide clustering due to the partial ice crystal formation in the solvent [11], can only be observed in the spectra of the bulk solution study but not in the nanochannel studies. This clustering of the n3 in the bulk solvent was previously reported [11] and demonstrated in a spin dilution experiment, which also showed that the spectral broadening could be significantly reduced with a decrease in the concentration of the spin-labeled n3 in the bulk solvent. Collectively, as indicated by the similarity of the P(r) distributions and the broadened cw-ESR spectral features, this study concludes that i) the n3β structure remains approximately (∼2.2 nm) unchanged over the studied conditions, and ii) the peculiar 3-nm peak is a composite result of the partial water crystallization within the solvent upon cooling to 50 K, the so-called cryoartifact.

### Determination of n3α Peptide Structure by DEER

The need for further confirming the structural integrity of n3 in our studied conditions is warranted since the DEER is performed at 50 K and the determined distances (Fig. 1) are along one leg of the n3β-d, even though the CD results in the previous study conducted at room temperature exhibit the same spectra as reported for the n3β. We therefore study the structure of n3α-d both in the bulk solvent and within the nanochannels at 50K.

Inset in Figure 2(a) shows a cartoon model of the n3α-d peptide. The time-domain DEER traces for the n3α-d in the (mostly) vitrified bulk solvent (blue) and in the MSU nanochannel (red) at temperatures 50 K (solid lines) and 70 K (dashed lines) were shown in Figure 2(a). The gray lines represent the exponential baselines that best fit the time-domain DEER data. The time-domain DEER signals displayed in Figure 2(a) were equally y-shifted for a clear presentation of the experimental data. Figure 2(b) reports the intra-molecular interspin distance distributions P(r), determined by the Tikhonov analysis [28], for the time-domain DEER data after the baseline removal. For the MSU nanochannel studies, the P(r) results resemble each other closely, indicating that the structure remains approximately the same over the temperature range. The most probable distance and the full-width at half-maximum (fwhm) of the peak are ca. 2.0 nm and 0.51 nm, respectively. The C_β_-C_β_ distance in the determined NMR structure [22] (PDB code: 1M25) of the n3α peptide is 1.1 nm, showing that the side chains of the two studied sites stretch out into the solvent in opposite directions from the helix axis (cf. Fig. 2a). Since the stretch length of one spin-label side-chain could be up to 0.7 nm, the determined distances (∼ 2.0 nm) of the present study are reasonably consistent with the structure of the NMR study. In the bulk solution studies, the P(r) results of 50 and 70 K are closely similar. The average distance is about 1.93 nm. As compared the P(r) results of the bulk solution to those of the MSU, they are slightly different wherein the former is clearly broader than the latter. The P(r) results of the bulk solution appear to have a small distance shoulder around 2.4 nm in addition to the major distance at 1.75 nm. The P(r) heterogeneity for the bulk solution study can be observed in different measurements at varying temperature. We attribute the presence of the broad and somewhat of heterogeneity P(r)s to the consequence of the solvent heterogeneity (caused by partial water crystallization; cryoartifact), as the fwhm of the P(r) can be easily compromised by the side chain disordering and the fact that the averaged distances of the P(r)s are about the same. This observation for the P(r) is also supported by the cw-ESR results (cf. Figure 2c) wherein the spectra display the existence of the multiple spectral components. The low-field peak in the cw-ESR spectra of the bulk solution studies clearly shows the evidence of multiple spectral components (as indicated by arrows), manifesting the existence of the local heterogeneity in the solvent when cooled to low cryogenic temperatures, thereby resulting in the peculiar peak around 2.4 nm in the P(r)s which can not be regarded as a ghost peak and removed in the DEER analysis. [37] This indicates that the inter-peptide distances are within the cw-ESR sensitive region, i.e. <2 nm, displaying a sign of molecular clustering in the bulk solution upon cooling. Collectively, the average distances of the n3α-d are close in all of the studied conditions and the structure of the n3α-d remains intact upon the encapsulation into the nanochannels, although the solvent heterogeneity does give rise to ambiguity in the determined P(r). This conclusion is consistent with the results of the n3β-d (cf. Figure 1) and supports our claims made in the end of last section.

### Interfacial Water Accessibility by ESEEM

To unravel the details of the local solvation of the encapsulated peptides in the nanochannels, this study carried out three-pulse ESEEM experiments at 50 K for [n3β-s] = 1.5 mM and [PPm3-s] = 1.5 mM in various local environments. ESEEM is a powerful technique to measure weak coupling between paramagnetic ions and nearby (<0.6 nm) nuclei with nonzero spin. [20], [30] Only magnetic nuclei that are weakly coupled to the spin label give rise to peaks in Fourier Transformed (FT)-ESEEM spectrum, centered approximately at the Larmor frequencies of the coupled nuclei. [38] Under the condition of strong coupling (i.e., A_iso_ ≥2ν_I_, where A_iso_ is the isotropic value of hyperfine tensors, and ν_I_ the nuclear Larmor frequency), it would lead to a splitting away from the Larmor frequency [39].

**Figure 3:**
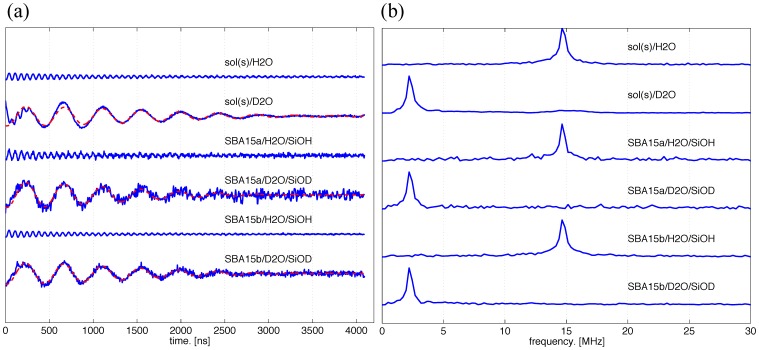
Water accessibility study of the n3β-s by ESEEM. (a) Three-pulse ESEEM time-domain data (solid lines) after the removal of the exponential decaying function in the raw data. The modulation depth is directly correlated to the peak intensity of the FT-ESEEM and can be quantitatively described by *k_D_* and obtained in the following analysis. The dashed lines represent the theoretical fits to the experimental data. According to ESEEM theory, a damped harmonic oscillation 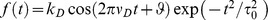, as a function of the ESEEM collection time *t*, can be employed. [20] The *f*(*t*) function was least-square fitted to the ESEEM time-domain data by varying the deuterium modulation depth, *k_D_*, damping constant, *τ_0_*, and phase, φ, while keeping the Larmor frequency of deuterium *ν_D_* fixed. The best-fit parameters are shown in Table 1. The signal-to-noise ratio of the experiments varies with many experimental conditions, such as instrumental setups, data collection time, and so on. Thus, FT-ESEEM has been a typical data presentation for the ESEEM analyses. (b) The FT-ESEEM data for the n3β-s in the studied conditions, including the vitrified bulk solvent and a variety of combinations of the nanochannel materials (SBA15a, SBA15b), solvents (H_2_O, D_2_O), and surface groups inside the materials (-SiOH or -SiOD).

Figure 3 shows (a) time-domain ESEEM and (b) the FT-ESEEM plots of the time-domain ESEEM for n3β-s. Note that the dashed lines in Figure 3(a) are theoretical fits to the experimental data and are to be discussed in the later paragraph. The studied conditions include a (mostly) vitrified bulk solvent and a variety of combinations varying nanochannel material (SBA15a or SBA15b), solvent (H_2_O or D_2_O), and surface groups inside the materials (-SiOH or -SiOD). The results clearly show that the peak position is dependent on the solvent molecules. As H_2_O is used as the solvent, the peak in the FT-ESEEM spectra is observed at frequency 14.7 MHz, which is approximately the same as the Larmor frequency of nucleus ^1^H. The peak in the FT-ESEEM spectra shifts to 2.2 MHz, which matches the Larmor frequency of deuterium, as D_2_O is used as the solvent. This demonstrates that the coupling between the nitroxide spin and the solvent molecules is a weak coupling.

In the bulk solution study of sol(s)/D_2_O (Fig. 3), the FT-ESEEM spectrum displays only the peak for deuterium but not hydrogen. This suggests that the solvent molecules within the ESEEM sensitive region (ca. <0.6 nm) near to the nitroxide spin are mostly D_2_O or a mixture of D_2_O molecules and OD of sucrose after the D/H exchange with the D_2_O bath. Note that the intensity of ^2^H in the FT-ESEEM representation is at least 20 times greater than that of ^1^H.

According to ESEEM theory, the modulation depth can be easily extracted from time-domain ESEEM signals, and is dependent on the number and distances of the nuclei coupled to the electron spin. The data can be immediately analyzed to yield a three-pulse ESEEM-based (deuterated) water accessibility parameter, Π(D_2_O), using Eq. (1), [19], [20]

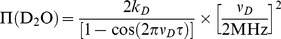
(1)where *k_D_* and *ν_D_* are, respectively, the modulation depth and Larmor frequency of deuterium, and *τ* the choice of the first interpulse delay. Note that the *k_D_* value can be precisely determined in a typical time-domain ESEEM analysis. [20] This Π parameter is sensitive to the presence of deuterium nuclei within the van der Waals contact distance of ∼0.35 nm from the spin label. The obtained Π values for the n3β-s are close (with small differences) to each other (cf. Table 1). Our finding shows that the water accessibility (Π) values of the spin-labeled site on the n3β-s are only slightly greater for the SBA15a than for the SBA15b and the bulk solvent.

**Table 1:** Parameters obtained in the analyses of the ESE and ESEEM data.§

	T_M_ (ns)#		x#		*C_ex_* (nm^−3^)	*k_D_* ¶	Π¶
	D_2_O	H_2_O	D_2_O	H_2_O	D_2_O	D_2_O	D_2_O
n3β-s-a	2950	1967	0.93	0.81	24.2	0.129	0.185
n3β-s-b	2156	1455	0.93	0.79	42.3	0.109	0.156
n3β-s-sol(s)	3545	2693	1.03	1.15	12.8	0.099	0.142
PPm3-s-a	2781	1785	0.82	0.81	28.6	0.151	0.215
PPm3-s-b	1004	703	0.93	0.74	60.9	0.092	0.132
PPm3-s-sol(g)	7687	3430	0.95	1.52	23.0	0.335	0.479
PPm3-s-sol(s)	5192	3496	1.20	1.45	13.4	0.160	0.229

§ Estimated errors: 5%(T_M_), 10%(x), 13% (*C_ex_*), 5% (*k_D_*), 10% (Π). Abbreviations: **n3-s-a** (the n3-s is within SBA15a containing pure water); **n3-s-b** (the n3-s is within SBA15b containing pure water); **n3-s-sol(s)** (the n3-s is in a vitrified bulk solvent containing 40 wt% sucrose, (s), in D_2_O or H_2_O); **PPm3-s-sol(g)** (PPm3-s is in a vitrified bulk solvent containing 40v/v% glycerol in H_2_O; deuterated glycerol is used if the solvent is D_2_O, a condition of which is represented by sol(dg)/D_2_O in main text); **PPm3-s-sol(s)** (PPm3-s is in a vitrified bulk solvent containing 40 wt% sucrose in D_2_O or H_2_O). In all of the experiments, the surface group of the nanochannels is modified to –SiOD in advance if D_2_O is used. See Method for details.

# The values of T_M_ and x are obtained in the analysis of the pulsed ESE measurements using a stretched exponential function, 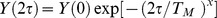, where τ is the time between the two pulses, x the exponent, and Y(0) is the echo intensity at τ  = 0. The obtained values are used to yield *C_ex_* using Eq. (2). The *C_ex_* represents ESE-based water accessibility within the range of ∼2 nm from the nitroxide spin.

¶ The *k_D_* values are obtained in the theoretical analysis of the ESEEM measurements as described in Figure 3. The best-fit values for the damping constant (*τ_0_*) and phase (φ) are very close together (2.9∼3.0). The Π represents ESEEM-based water accessibility within the range of ∼0.35 nm from the nitroxide spin.

**Figure 4:**
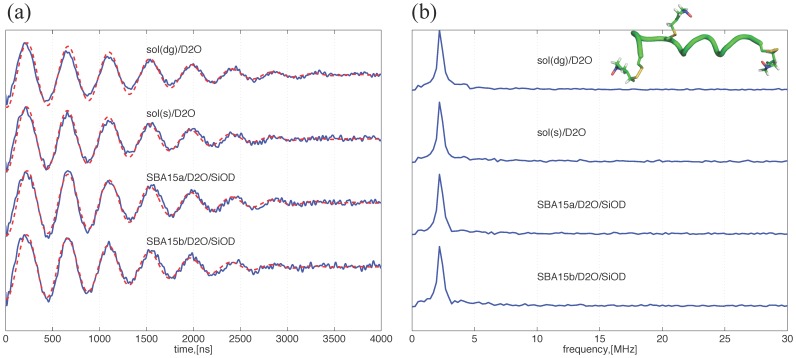
Water accessibility study of the PPm3-s by ESEEM. (a) Three-pulse ESEEM time-domain data (solid lines) after the removal of the exponential decaying function in the raw data. The modulation depth is directly correlated to the peak intensity of the FT-ESEEM and can be quantitatively characterized by the best-fit parameter *k_D_* (cf. Table 1). The dashed lines represent the theoretical fits to the experimental data using the equation described in Figure 3. (b) The FT-ESEEM data for the PPm3-s in various deuterated conditions. The peaks correspond to the Larmor frequency of nucleus ^2^H, indicating the PPm3-s is surrounded by D_2_O. The inset shows a ribbon model of a PPm3 variant carrying three spin labels.

Additionally, this study carries out the ESEEM measurements for the PPm3-s in various conditions as shown in Figures 4(a) and (b) for the time-domain versus FT-ESEEM, respectively. Two of the PPm3 variants (PP-3R1 and PP-2R1; see [23]), which are of the same length as PPm3 but differ in spin-labeling sites, were previously studied and demonstrated to possess a PPII helical structure in SBA15a, MSU, and in sol(s)/H_2_O. The inset of Figure 4(b) shows the structure of the triply labeled PPm3 variant. In the present study, pure D_2_O was used as the solvent and the inner surface of all the nanochannels was modified to be deuterated silanol groups in the measurements. The FT-ESEEM results clearly show the presence of the Larmor frequency of ^2^H. Although the PPm3-s (in the sol(s)/D_2_O) is supposed to be surrounded by a mixture of –OD and –OH, the signal corresponding to ^1^H is not observed in the measurements. This is consistent with the observation for the n3β-s (cf. sol(s)/D_2_O in Figure 3). By theoretical analysis (Eq. 1), the *k_D_* and Π values can be extracted from the ESEEM measurements and used to determine the contributions solely from the deuteron. As shown in Table 1, except for the result of the PPm3-s with sol(dg)/D_2_O, the values for the PPm3 studies are close to those obtained for the n3 studies and are within the typical range for a solvent-exposed site on a protein (discussed later). The differences in the Π values between the studies of the two peptides are small and are considered to reflect only the minor differences in the respective local solvation environments. Whereas, the *k_D_* and Π values of the PPm3-s in sol(dg)/D_2_O are at least two times greater than the others for the PPm3 (i.e. those without the deuterated cryoprotectants or without cryoprotectant at all in the nanochannel cases; cf. Fig. 4). Apparently, the deuterated glycerol molecules play an important role in the ESEEM signals and consequently the *k_D_* and Π values. Therefore, this study shows that the water accessibility of the hydration layer at the interface with the peptides (within the ESEEM sensitive region) in the bulk solvents is at least two times greater than that in the nanochannels.

This analysis method was usefully demonstrated for obtaining a reliable water accessibility parameter for each of the spin-labeled sites in a large transmembrane protein in deuterated water. [20] Previously, Π value was found to be 0.13∼0.25 for typical solvent-exposed sites on a protein, below 0.1 for buried residues, and >0.35 for unbound spin labels. [20] The obtained Π values of the present study (except for the sol(dg)/D_2_O) are within the range for the solvent-exposed sites. They are distinctly greater than the values for a typical buried site of a protein, and less than the values for an unbound spin label. Our study indicates the following: i) the local solvation changes little with the secondary structures (n3β-s versus PPm3-s) as well as nanochannel sizes, and ii) the deuteron accessibility within the range ∼0.35 nm from the spin label in the nanochannels is roughly half the value for the peptides immersed in the vitrified bulk solvents.

### Interfacial Water is Non-crystalline

The interfacial water is expected to be amorphous (either liquid or solid) due to the rapid cooling scheme employed here, and the added cryoprotectants in the bulk case or the nanoconfinement in the nanochannel study. To confirm this expectation, this study also performed ESEEM measurement for the n3β-s (and DEER for the n3β-d) in pure water at 50 K, and found that no ESEEM (and DEER) signals were obtained, because of the formation of crystalline ice at 50 K, which resulted in a significantly shorter T_M_ value and the rapidly decaying ESEEM (and DEER) echo signals as nitroxide is known to form hydrogen bonds with water. (T_M_ represents spin phase memory time.) In the presence of largely crystallized ice, the hyperfine coupling would become much stronger due to the formation of the hydrogen bonding to nitroxide. In such a case, electron nuclear double resonance (ENDOR) experiment is a much better choice than ESEEM as ENDOR is proven to be sensitive to strong hyperfine couplings. [40] This observation adds evidence to support the non-crystalline nature of the surface water and is consistent with earlier reports from neutron scattering and NMR experiments [2], [41].

Moreover, the ESEEM data is also informative for identifying the hydrogen-bonded versus non-bonded water molecules to the N-O group of the spin label in a partially frozen state. Previous studies [38] by DFT calculations and experiments showed that water molecules that participated in a moderate hydrogen-bonding to the N-O radicals (i.e. a condition of partially crystalline state) resulted in a broad (>±2MHz in half-height width) spectral component in FT-ESEEM data, while the non-bonded water molecules gave rise to a narrow (<±0.5MHz) spectral component centered at Larmor frequency of hydrogen (or deuterium). The two spectral components would coexist in a partially frozen state around a nitroxide. [38] It is evidently shown (Figures 3b and 4b) that all of the FT-ESEEM spectra is characterized by one single narrow component, suggesting the spin label is surrounded by the water molecules that are in van der Walls contact with the nitroxide spin but are not in the correct orientations for hydrogen bonding; the spectra would otherwise become much broader. No broad component was found as the inter-pulse delay was decreased from 400 to 100 ns to probe the fast-decaying component in our study. This observation suggests the surface water remains non-crystalline/amorphous in the nanochannels (also in the vitrified bulk solvent) at 50 K. This also adds evidence to the aforementioned result concerning the non-crystalline surface water.

### Long-Range Water Accessibility for the Nitroxide Spin by Pulsed ESE

**Figure 5:**
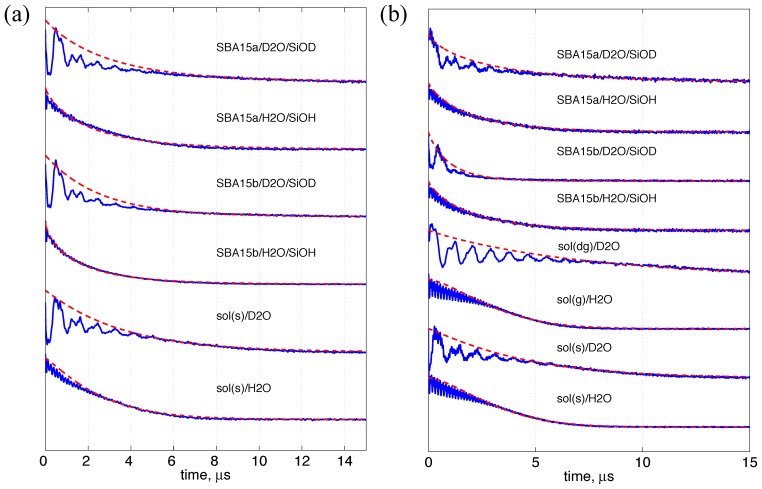
Long-range water accessibility study by ESE. The theoretical fits (red lines) to the ESE experimental data (blue lines) using a stretched exponential function (see Table 1) as previously described. [11] The results for the n3β-s and PPm3-s are shown in (a) and (b), respectively. The decay signals acquired by the ESE experiments were fitted over the maxima of the deuterium modulation as described in Zecevic et al. [32] to minimize the influence from destructive interference of nuclear modulations. The obtained values of the T_M_ (in ns) and stretching exponent x are shown in Table 1. The T_M_ values can be directly used to yield the surrounding proton density (*C_ex_*; cf. Eq. 2) within the range of ∼2 nm from a nitroxide spin.

Quantification of water accessibility at a somewhat larger distance range (ca. 2 nm from the nitroxide spin, which would cover several water layers) can be obtained from the analysis of the difference in the 1/T_M_ values of the ESE measurements between non-deuterated and deuterated water molecules. This difference provides a reliable estimation for the concentration of exchangeable protons, *C_ex_*, within the range of ∼2 nm from a nitroxide spin. The concentration can be yielded by Eq. (2), [20]

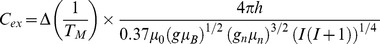
(2)where *I* is the nuclear spin quantum number, *µ_n_* the nuclear magneton, *µ_B_* the Bohr magneton, *g_n_* and *g* the nuclear proton and electron *g* values, respectively. Figure 5 shows the analyses of extracting T_M_ from the ESE measurements of the two peptides, n3β-s and PPm3-s. See Table 1 for the obtained T_M_ values (in a unit of ns) as well as the *C_ex_* values (cf. Eq. 2). Note that for the bulk solvent studies of PPm3-s, the *C_ex_* extracted from the data of sol(dg)/D_2_O versus sol(g)/H_2_O is two times greater than those for sol(s)/D_2_O versus sol(s)/H_2_O. This is because that the contribution from the sucrose is absent in the *C_ex_* analysis since the deuterated sucrose in not conveniently available to the present study. Thus, we regard *C_ex_*  = 23.0 (i.e., the one extracted from the PPm3-s in sol(dg)/D_2_O versus sol(g)/H_2_O) as a genuine value representing the local solvation of the both peptides in the bulk vitrified solvent. The *C_ex_* value for the bulk solvent is slightly less than that of the SBA15a. As compared to a proton concentration in pure water of 67 nm^−3^ (i.e., 1 g/cm^3^), the obtained values are approximately half and are reasonable, since the spin-label site on the peptides should only experience fewer than a half number of the water molecules surrounding the peptide. The only exceptions are the results of the n3β-s and PPm3-s in SBA15b (*C_ex_* = 42.3 ∼ 60.9), indicating that the two peptides in this case experience higher water densities. (Such a high *C_ex_* value for a spin-labeled peptide/protein in a bulk solvent has never been reported.) These ESE results indicate that the density of exchangeable protons within the range of ∼2 nm from a nitroxide spin is ordered as follows: SBA15b>SBA15a>bulk solvent. In particular, the results of the nanochannels indicate that the proton density increases substantially with the decrease in the nanochannel pore size. It is appropriate to briefly comment on the relevance of this result to other studies. Previous computational studies [42], [43] have shown that the water density is lower within the center of the nanochannel than close to the inner surface of the nanochannel. These studies, along with other experimental works, evoke a reasonable explanation for the higher water density in SBA15b than in SBA15a observed in the present study, as the nitroxide spin-hosting surface of the n3β-s and PPm3-s peptides and the inner surface of the nanochannel come closer to each other in SBA15b than in SBA15a. Further discussion is presented below.

## Discussion

### An Intrinsic Property of the n3 peptides

The 14-mer-long polyproline model peptide was previously demonstrated by DEER (50 K) that it remained in a PPII conformation in the nanochannels and in the vitrified bulk solvent (sol(s)/H_2_O), and that the P(r) of the polyproline model peptide results showed no evidence of peculiar peaks. [23] Moreover, the corresponding cw-ESR spectra of the polyproline model peptides showed no sign of large spectral broadening as this study reported for the n3 peptides (cf. Figures 1c and 2c). These DEER and cw-ESR experiments were measured in the same solvent/experimental conditions except the peptide types (PPm3 variants versus n3). Therefore, we think that it is an intrinsic property of the n3 peptide that tends to being spatially clustered (to a moderate extent) in the studied bulk solvent. This observation enhances the conclusion made for the results of n3α-d and n3β-d in the present study, providing an explanation for the peculiar minor peaks observed in Figures 1(b) and 2(b).

### Close Relevance of the Cryogenic Studies to the Conditions at Room Temperature

Before delving into the implications of the experimental data, we discuss the relevance of the findings at 50 K to our understanding toward the room-temperature phenomena, which are also concerned. It is generally presumed in the protein crystallography community that room-temperature conformations of proteins, along with the structures of their surrounding water, can be preserved even at cryogenic temperatures as long as the protein crystals are cooled fast enough, as rapid cooling would leave the molecules no time to move around or wiggle considerably. Nevertheless, the regular practices in cryo-cooling protein crystals hardly, if not impossibly, attain the optimal cooling rates, and the structures observed at cryogenic temperatures turn out to be a snapshot of the structures at much lower temperatures, even down to 200 K, [44] therefore casting doubt on the biological relevance of the observations made at cryogenic temperatures. The limits on achieving the optimal cooling rates may plausibly result from the sheer sizes of typical protein crystals and the cooling method. With the common crystal size of 0.2×0.2×0.2 mm and the ordinary flash-cooling method (placing crystals directly under cold gas stream), cooling to 100 K may need the time scale of 10^−1^ seconds to reach thermal equilibrium. [45] Fortunately, our peptide-trapped-in-nanochannel samples are in the form of powder, which is equivalent to a collection of very tiny crystals, in the length scales orders of magnitude smaller than those of typical protein crystals. Due to the much-reduced sizes of the crystallites, combined with the more efficient plunge-cooling method employed here, the cooling rate for our samples are expected to be orders of magnitude higher. [45] This study thus expects that the peptide conformation and the water molecular distribution observed in the nanochannel studies faithfully reflect the conditions at room temperature. Indeed, the P(r) obtained from the DEER measurements at 50 K agrees with the NMR results collected by Kozin *et al.* [21] at room temperatures. Even in the bulk solution study, where the sample volumes are larger, the concerned aspects of our experimental results (i.e., backbone structure of the peptide and the interfacial water density) should still be relevant. Comparisons between the protein structures solved at room temperature and at cryogenic temperatures have demonstrated minimal cooling-induced rms deviations, within 0.08 nm, in the protein backbones. [46], [47] A careful ESR study conducted very recently has demonstrated that cooling rate, or even the very act of cooling itself, may have no observable effect on protein structural properties, manifested as the main P(r) peak positions for four different pairs of spin-labeled residues of T4 lysozyme in bulk solutions. [48] Based on the rationales reasoned above, we have considerable confidence in the room-temperature relevance of our data, even though they were collected at 50 K. This allows us to compare the experimental results with room-temperature observations from the literature and make contributions to the understanding of the room-temperature phenomena involving water.

### Density Heterogeneity of the Confined Water Surrounding the Peptides

The present study has reported the ESE-proven correlation between the nanochannel diameter and the water accessibility within 2 nm from the nitroxide spin. Here, we shall discuss its physical origin and implications. The P(r) results show that the intra-molecular interspin distance of the n3β-d peptide is ∼2.2 nm, regardless of the nanochannel size (Figure 1b). Given this distance and the relative positions of the two spin-labeled sites (Figure 1a), it is reasonable to expect an equivalent diameter of >2.2 nm for freely rotating n3β peptide. Assuming that diameter is ∼2.5 nm and the peptide is located near the nanochannel center, the distance between the peptide surface and the channel wall would be ∼2.55 nm for SBA15a (with the diameter of 7.6 nm) and ∼1.8 nm for SBA15b (with the diameter of 6.1 nm). Since the nitroxide spin is ∼0.7 nm from the n3 peptide surface, the range covered by the ESE measurements in assessing the water accessibility should be ∼2.7 nm from the peptide surface, encompassing all the relevant space between the peptide and the channel wall for both of the SBA15a and SBA15b cases. In other words, the density of the exchangeable protons (presumably from water molecules) extracted from the ESE measurements is in fact the water density averaged over the entire inter-surface space. It has long been established from experimental and computational studies that water in the first hydration layer (∼0.3 nm thick) of a surface exhibits a density distinctly greater than that of bulk water, due to the perturbations on water by that surface. [49]–[51] Since this surface effect is always present and independent of the nanochannel diameter so long as the inter-surface space does not shrink to a few water layer thick, the amount of the bulk-like water halfway between the two surfaces would gradually diminish when the nanochannel diameter keeps decreasing, allowing the surface water to become dominant and the averaged water density to rise. This explains why the averaged water density within 2.7 nm from the peptide surface is greater in SBA15b than in SBA15a. Furthermore, given the extent of the density elevation (at least about two times) upon adopting SBA15b (see the previous section) and the fact that water accessibility within 0.35 nm from the nitroxide spin is of the same magnitude between SBA15a and SBA15b (see the results from the ESEEM measurements; Table 1), we argue that water layers beyond the first hydration water also exhibit higher-than-bulk-water densities and contribute to the observation in the ESE measurements. Since the inter-surface space for SBA15b is ∼1.8 nm thick if the peptide exhibits an equivalent diameter of ∼2.5 nm (see above), we speculate that the peptide surface and the nanochannel wall may each perturb up to 3 layers or 0.9 nm thick of water from the surfaces (i.e., 2×3×0.3 = 1.8 nm), leading to the higher-than-bulk-water densities in these layers. Indeed, earlier experimental and computational studies indicate that the influence of the surface effect is well beyond the first hydration layer [52]–[54] and probably up to 1 nm from the surface, as indicated by a femtosecond-resolved fluorescence study [53]; Kim *et al.* [54] even went as far as classifying the 3 surface-perturbed water layers as one layer of “well-ordered rigid water” and two layers of “quasi-bound water”. Our study reports one of the few experimental, albeit indirect, evidence in the context of water density to support the existence of multiple (more than one) surface-perturbed water layers, and may provide a foundation to investigate the structural origin of the “dynamic” hydration layer proposed by Zewail’s and Zhang’s groups [53]–[55].

Support to the above speculation can also be gained by examining the ESE results of the PPm3-s. Previously it was shown that this long helical polyproline-based peptide remains structurally intact and undergoes a large degree of rotational anisotropy and orientational ordering inside the SBA15 nanochannels. [23] The PPm3 was demonstrated to align favorably with its long axis being parallel to the longitudinal axis of the nanochannels. The present study reports that the *C_ex_* values are 60.9 and 28.6 for SBA15b and SBA15a, respectively, showing a good agreement with the finding for the n3β-s. Both the peptides, though in two different secondary structures, report a similar trend for the *C_ex_*, suggesting the water density variation along the transverse direction of the nanochannel is the key to the observed changes in the ESE measurements and could be independent of the topological details of a surface. Besides, the present study verifies the density heterogeneity along the peptide surface normal in the nanochannels.

The results from the ESEEM measurements tell another interesting story. The ESEEM study reports the water accessibility within the van-der-Waals contact of 0.35 nm from the nitroxide spin; this range corresponds to the first hydration water of the peptides. The Π values for the two peptides in the nanochannels with different diameters are roughly the same and within the range for a typical solvent-exposed site on a protein. This indicates that the first hydration water accessibility is essentially unchanged (within errors) in nanospace, irrespective of the peptide secondary structure, peptide length, or the nanochannel diameter. This fact further enhances our argument in the previous two paragraphs that the surface effect is unaffected by the nanochannel size. In a comparison of the bulk and nanochannel studies, the results of the Π analysis are similar if sucrose is used. However, the Π value for the bulk solution becomes double if deuterated glycerol is used. As shown in Eq. (2), the Π value is not affected by the non-deuterated cryoprotectants. Therefore, this ESEEM study manifests that the interfacial hydration accessibility (i.e., the first layer) for the bulk solvents is approximately two times greater than that in the nanochannels, while the secondary structure remains approximately unchanged. This suggests that variations in the interfacial solvation density are not of considerable influence to the stability of the peptide structures. The present finding could support the concept that presence of the first hydration layer, in contrast to the long-held belief, is a sufficient but not indispensible condition for ensuring the proper function and structure of a protein, as demonstrated in two previous studies that i) a completely unsolvated polyalanine helix in the gas phase could remain up to at least 723 K; [7] ii) water may even be replaced with a polymer in rendering a protein the dynamical flexibility and biological functionality. [56] Taken together the present and the previous studies, we argue that the first hydration layers might play a role in loosening and providing flexibility to the structure of a protein rather than stabilizing it.

Altogether, this appears to indicate that the first hydration water accessibility is independent of the surface topography of the peptide; the spatial arrangement of the water molecules in the first hydration layer has no considerable impact on the peptide conformation. Moreover, the first hydration water accessibility seems to be unaffected by the condition of the bulk water, because this accessibility (Π values) changes little as compared to the SBA15a and SBA15b, where the bulk water for the latter is considerably diminished or even absent. All these observations evoke the need of a picture alternative to that involving the bulk water effect on hydration water, as proposed by Merzel and Smith [50], to explain how the surface topography affects the first hydration water density. How much the surface topography affects the first hydration water density is even a matter of debate in lieu of our ESEEM data.

### Inter-peptide Interactions are Excluded from the Analyses

This study shows that given the same [n3β-d] concentration, the baseline of the DEER measurement for the SBA15b decays rapidly as compared to the baselines of the SBA15a and bulk solution studies (Figure 1a). This indicates the bulk spin concentration (denoted as [n3β-d]′ hereafter) is increased upon the encapsulation into the SBA15b, suggesting the spatial distribution of the n3β-d peptides is adjusted for the nanochannel structures. The apparent spin concentration, i.e., [n3β-d]′, in the SBA15b, if too high, would likely give rise to a greater degree of the unwanted inter-spin interactions (e.g., instantaneous diffusion, which becomes important as spin concentration is sufficiently high,) and the n3β-d peptide clustering/aggregation in the nanochannels. However, the following summary of the results indicates that the two suspected conditions hardly exist in this study. Cw-ESR lineshape is sensitive to inter-spin distances less than 2 nm. [57] The cw-ESR spectra (Fig. 1c) of the SBA15a and SBA15b are similar to each other and show no sign of the inter-peptide dipolar broadening. (Same observation can be obtained from the n3α-d study shown in Figure 2c.) This indicates that the inter-peptide distances in the nanochannels are long enough (>2 nm) so that the inter-molecular dipolar interactions are too weak to appear in the cw-ESR spectra. To investigate whether or not the instantaneous diffusion plays an important role in the nanochannel study, we thus performed the DEER measurement with increasing the length of the observer π/2 and π pulses from 16 and 32 ns, respectively, to 32 and 64 ns, respectively. If the instantaneous diffusion plays an important role in the nanochannel studies, we would observe an increase in the signal-to-noise ratio (SNR) of the DEER data with the increased pulse length. This is because of that as the instantaneous diffusion is important, excitation of a larger fraction of spins may not necessarily translate to higher sensitivity. [58] Our measurement found that no increase in the SNR was observed in the experiment. Taken together, the above observations rule out the two suspected conditions. The individual peptides are sufficiently separated (>2 nm) in both the SBA15a and SBA15b nanochannels. The observed increase in the proton density for SBA15b is indeed attributable to the increased solvent (proton) density that results from the decrease in the nanochannel pore size. This study strongly suggests that the analysis results of the ESEEM and ESE measurements for the nanochannel studies reflect largely the difference in the solvation shells (i.e., the interactions between the nitroxide spin and the nearby solvent molecules,) rather than the inter-peptide interactions.

Last, we comment on the effect of the increased [n3β-d]′ on the P(r) determination of the SBA15b. Previously, the n3β-d was studied by the DEER technique at 50 K, with [n3β-d] = 1.5 mM (which is three times greater as compared to the present study) in the nanochannel of the MSU material. [11] The P(r) obtained in the previous study is closely similar to the distance distributions of the SBA15a and SBA15b in the present study. This observation indicates that the variation of the bulk spin concentration affects insignificantly the determination of the distance distribution by Tikhonov regularization method. The use of nanochannel is useful to avoid clustering of spin-labeled molecules, making it possible to perform DEER in higher concentrations.

### Conclusions

In summary, we have studied two different spin-labeled peptides in the (mostly) vitrified bulk solvents and in the nanochannels of several different pore diameters (6.1 ∼ 7.6 nm) using ESR techniques (cw, DEER, ESEEM, and ESE) at 50 K. The studied peptides include a 26-residue-long polypeptide in two conformations (α-helix versus β-hairpin), inducible by the change of the solvent, and a 14-residue-long polyproline model peptide. This study demonstrates the peptide being confined but structurally unaltered in the nanospace, with its surrounding water exhibiting density heterogeneity along the peptide surface normal. The DEER measurements demonstrate that the peptide conformations remain approximately unchanged upon the encapsulation into the nanochannels. The ESEEM results show that the water accessibility in the immediate vicinity of the nitroxide label (the first hydration layer, within the van der Waals contacts, ∼0.35 nm) at the water-peptide interface changes little with the nanochannel dimensions but reduce by half as comparing to the accessibility obtained for the bulk solvent studies. Nevertheless, the ESE results show that the hydration density in the range of ∼2 nm from the nitroxide spin (or ∼2.7 nm from the peptide surface) varies notably and increases with the decreasing nanochannel diameter; this is considered to be a consequence of diminishing or completely removal of the bulk-like water when the nanochannel size is reduced. Indeed, the ESE shows the long-range water accessibility in the SBA15a nanochannel (i.e., of the largest pore size in this study) changes little as compared with the bulk solvent. Our conclusions are based on the observed relative changes of water density among different confinement conditions at a constant temperature of 50 K; therefore, the changes upon temperature variations (e.g., abrupt changes of water properties accompanying phase transition upon cooling) are irrelevant to the validity of our conclusion. We infer from these observations that i) the water accessibility of the first hydration layer appears to be essentially unchanged, regardless of the water properties outside the first hydration layer or the surface topological details of a peptide; ii) perturbations on water, induced by the peptide and nanochannel inner surfaces, may each affect up to several (2∼3) layers of water from these surfaces, and is independent of the surface topological details, too. This second conclusion experimentally demonstrates the range of the surface effect on the surrounding water, while the first conclusion might evoke the need of alternative insight to explain why water exhibits density variation across the protein surface to the bulk. Moreover, this study shows the polypeptides are confined, but free and hydrated in nanochannels. The secondary structures of the peptides appear to be insensitive to the solvation properties such as the solvation accessibility, as this study (which is solely based on the studies of the n3 and PPm3 peptide variants) demonstrates the peptides retain their structures even the solvation accessibility is changed by a large extent. This finding suggests that the density property of the interfacial solvation (namely, the solvation in the immediate vicinity of the spin-labeled sites) is not essential to the stability of a peptide structure. This study has opened up exciting avenues for looking into the protein-water interactions and the biomolecules in nano-confinements using ESR with nanochannels.
